# Mental health, insomnia and suicidal ideation during treatment with apremilast

**DOI:** 10.1111/ajd.13864

**Published:** 2022-05-16

**Authors:** Timothy L. Cowan, Anna Wilson, Dedee F. Murrell

**Affiliations:** ^1^ Department of Dermatology St George Hospital Sydney Australia; ^2^ Faculty of Medicine UNSW Sydney Australia

**Keywords:** apremilast, insomnia, mental health, phosphodiesterase‐4 inhibitor, suicidal ideation

The efficacy of apremilast, a phosphodiesterase‐4 inhibitor, in the treatment of psoriasis has been established.^1^, ^2^, ^3^ We report on two patients who developed psychiatric symptoms since the commencement of apremilast.

Patient A, a 38‐year‐old man with whole‐body psoriasis, was commenced on apremilast. He had previously completed treatment for latent tuberculosis 1 year prior to apremilast. Over a two‐week period, he titrated to a dose of 30 mg twice daily. Upon reaching this dose, he developed insomnia and, subsequently, suicidal ideation. He continued apremilast despite these symptoms with the 6 month supply provided to him in accordance with the Australia Medicare allowance and did not contact the prescriber or seek medical attention. He had nocturnal suicidal ideation for 3 weeks, and this was then resolved without treatment. He did not have any suicide attempts or plans and did not inform his partner of these thoughts. He continued to take his apremilast during this period. Despite resolution of suicidal ideation, he continued to have insomnia with functional impairment during the daytime and self‐ceased apremilast 4 months after commencement. Resolution of insomnia occurred within 2 weeks of cessation. He reported improvement of his psoriasis while on apremilast however had relapsed at the time of review due to self‐cessation.

Patient B, a 45‐year‐old woman with whole‐body psoriasis, was commenced on apremilast. She had a history of mild depression managed on a stable dose of paroxetine for 7 months prior to apremilast. She otherwise had a history of osteopenia and insulin resistance with no medication changes within a year. Within 2 weeks of commencement on apremilast, she developed insomnia and subsequent nausea and headaches. She self‐ceased treatment due to these side effects and had complete resolution within a week of cessation.

Apremilast is an effective and relatively safe option for the treatment of mild‐to‐moderate psoriasis.^1^, ^2^, ^3^ Insomnia or suicidal ideation was not reported as an adverse event in two pivotal randomized controlled trials;^2^, ^3^ however, patients with a history of depression are excluded from all clinical trials. Insomnia has been reported in 11% of 40 patients in a small observational study.^4^ Reports of suicidal ideation due to apremilast are rare,^5^ and no difference was reported against placebo in the randomized‐controlled trials.^2^, ^3^ The significance of this symptom, in addition to the knowledge that depression, is a known comorbidity of psoriasis, justifies close monitoring of patients on apremilast.^1^

Due to the emergence of these psychiatric symptoms in patients with no history of mental illness or other obvious risk factors, it is suggested that the risk of these symptoms is carefully explained on commencement and a mental health screen is performed at each review during their treatment course. We suggest that patients be given a 2‐month supply initially and then be reviewed rather than the full 6‐month supply available on Medicare in Australia. We also suggest that follow‐up consultations are conducted more frequently than what is currently required by Medicare in Australia.

## FUNDING INFORMATION

Nil.

## CONFLICT OF INTEREST

Nil.

## PATIENT CONSENT

Patient consent was gained for use of the case for publication.
